# Epicardial ventricular ablation in high risk patients

**DOI:** 10.1186/1749-8090-8-S1-O57

**Published:** 2013-09-11

**Authors:** G Coronella, L Argenziano, P Oliviero, A Contaldo, F Petteruti, G Pescatore, S Giordano, A La Marca, P Pepino, S Nardi

**Affiliations:** 1 Department of Cardiothoracic Surgery, Presidio Ospedaliero Pineta Grande, Castel Volturno, Italy; 2 Department of Cardiology, Presidio Ospedaliero Pineta Grande, Castel Volturno, Italy; 3 Department of Anaesthesiology and Intensive Care, Presidio Ospedaliero Pineta Grande, Castel Volturno, Italy

## Background

Catheter ablation is one of the options for the treatment of ventricular tachycardia (VT). In patients who have had a failure of the endocardial ablation (symptoms or continuous device schok) a surgical epicardial ventricular ablation is indicated via antherior thoracotomy or median sternotomy, according to the location of the focus.This paper describes our experience using this technique.

## Methods

From July 2011 up to May 2013, 10 consecutive patients, with a story of previous endocardial ablation and ICD were admitted for arrhythmic storm or palpitations or continuous shock. All patients were male, mean age 66,28 ± 6,4, left ventricular ejection fraction 35% ± 8,9%. The procedure was performed via anterior left thoracotomy. A surgical exposition of the femoral artery and vein was performed to grant a useful access for extracorporeal circulation. In two cases the procedure was done in left ventricular assistance. The mapping and the ablation of the left ventricle was done, first in sinus rhythm and in two patients in induced ventricular tachycardia using a 4 mm radiofrequency catheter on the epicardial surface of the heart while the tridimensional reconstruction was achieved in sinus rhythm using the “CARTO 3D mapping system” (Biosense). The apex was ablate in all patients creating an encircling line around the apex using a radiofrequency unipolar catheter.

## Results

There were observed not major complications. During the hospital stay all the patients were monitored and no one showed a ventricular tachycardia or malignant arrhythmia. The discharge happened 4,6 ± 1,2 days after the procedure with a hospital stay of 9,3 ± 3,14 days.

## Conclusion

The epicardial ablation is an effective strategy for patients in whom the endocardial ablation failed, with good results at the discharge from the hospital. Direct visualization of epicardial structures, catheters, and lesions may improve the safety and efficacy of epicardial catheter ablation.

